# Defining Bedaquiline Susceptibility, Resistance, Cross-Resistance and Associated Genetic Determinants: A Retrospective Cohort Study

**DOI:** 10.1016/j.ebiom.2018.01.005

**Published:** 2018-01-09

**Authors:** Nazir A. Ismail, Shaheed V. Omar, Lavania Joseph, Netricia Govender, Linsay Blows, Farzana Ismail, Hendrik Koornhof, Andries W. Dreyer, Koné Kaniga, Norbert Ndjeka

**Affiliations:** a National Institute for Communicable Diseases, Centre for Tuberculosis, Johannesburg, South Africa; b Department of Medical Microbiology, University of Pretoria, Pretoria, South Africa; c Janssen Research & Development, Titusville, NJ, United States; d National Department of Health, Tuberculosis Control and Management Cluster, Pretoria, South Africa

**Keywords:** Tuberculosis, Bedaquiline, Clofazimine, Mic, Mutation

## Abstract

**Background:**

Bedaquiline (BDQ) is a novel agent approved for use in combination treatment of multi-drug resistant tuberculosis (MDR-TB). We sought to determine BDQ epidemiological cut-off values (ECVs), define and assess interpretive criteria against putative resistance associated variants (RAVs), microbiological outcomes and cross resistance with clofazimine (CFZ).

**Methods:**

A retrospective cohort study was conducted. Minimal inhibitory concentrations (MIC) to BDQ were determined using 7H9 broth microdilution (BMD) and MGIT960. RAVs were genetically characterised using whole genome sequencing. BDQ ECVs were determined using ECOFFinder and compared with 6-month culture conversion status and CFZ MICs.

**Findings:**

A total of 391 isolates were analysed. Susceptible and intermediate categories were determined to have MICs of ≤ 0.125 μg/ml and 0.25 μg/ml using BMD and ≤ 1 μg/ml and 2 μg/ml using MGIT960 respectively. Microbiological failures occurred among BDQ exposed patients with a non-susceptible BDQ MIC, an *Rv0678* mutation and ≤ 2 active drug classes. The *Rv0678* RAVs were not the dominant mechanism of CFZ resistance and cross resistance was limited to isolates with an *Rv0678* mutation.

**Interpretation:**

Criteria for BDQ susceptibility are defined and will facilitate improved early detection of resistance. Cross- resistance between BDQ and CFZ is an emerging concern but in this study was primarily among those with an *Rv0678* mutation.

## Highlights

• Criteria for BDQ susceptibility are defined which will facilitate improved early detection of BDQ resistance
• Microbiological failures occurred in those with a non-susceptible BDQ MIC, an Rv0678 mutation and ≤ 2 active drug classes
• Cross resistance between CFZ and BDQ was primarily observed in patient isolates with *Rv0678* mutants

This study robustly defines susceptibility criteria for bedaquiline and addresses uncertainties around the genetic basis of resistance and cross resistance. Susceptibility criteria are provided for MGIT 960 and broth microdilution. Our findings do not support earlier studies indicating a role for selected putative genes (*pepQ* and *Rv1979*) in conferring resistance whilst clarifying the role of mutants in the *Rv0678* gene. Cross resistance with clofazimine was limited to cases with an *Rv0678* mutation and bedaquiline exposure. Furthermore, unlike in-vitro selected mutants where *Rv0678* mutation are the dominant cause of CFZ resistance, this does not appear to be true for clinical isolates.

## 1 Introduction

Drug-resistant tuberculosis (TB) has been declared a public health “crisis” by the World Health Organization (WHO, 2014), demanding drastic action. Bedaquiline (BDQ), a novel class of anti-mycobacterial drug specifically inhibiting mycobacterial adenosine triphosphate synthase (Preiss et al., 2015, Koul et al., 2007), was approved by the United States (US) Food and Drug Administration (FDA) in 2012 for the treatment of multi-drug resistant (MDR-) TB (Cox and Laessig, 2014), as part of combination therapy. Introduction of this novel agent to MDR-TB management has been a major advancement, reducing time to sputum culture conversion and improving patient outcomes (Ndjeka et al., 2015, Guglielmetti et al., 2015, Udwadia et al., 2014, Diacon et al., 2009, Diacon et al., 2014). South Africa is one of the leading countries to scale up use of this new drug under programmatic conditions with over three thousand cases initiated on a BDQ based regimen between 2015 and 2016, following the WHO Interim Policy Guidance on its use in MDR-treatment (WHO, 2013). The need to monitor minimal inhibitory concentrations (MICs) of local clinical isolates to the drug as an interim measure was recommended, since provisional criteria defining resistance were not globally adopted.

Two studies have attempted to define phenotypic criteria for resistance to BDQ (Torrea et al., 2015, Keller et al., 2015); however these were either conducted on a small number of relevant isolates or employed imprecise methods. The European Committee on Antimicrobial Susceptibility Testing (EUCAST) has set a provisional clinical breakpoint of ≤ 0.25 μg/ml irrespective of test method. This is a limitation as phenotypic testing of *Mycobacterium tuberculosis* (*Mtb*) is performed using different methodologies, which require method specific criteria as is observed for other drugs (WHO, 2012). In contrast, the United States Food and Drug Administration (FDA) agency has recommended MIC testing and has reserved a decision pending more data being available. The increasing use of BDQ and reports of clinical failures (Bloemberg et al., 2015, Hoffmann et al., 2016) necessitates robust data to develop criteria in aiding early detection of resistance and rapid introduction of appropriate control measures (Salfinger and Migliori, 2015, Veziris et al., 2017).

Great interest has also emerged on identifying the genetic targets associated with BDQ resistance to aid the development of rapid molecular tools. Although the *atpE* gene which encodes the BDQ target ATP synthase is biologically linked to resistance in vitro (Segala et al., 2012), mutations in this gene that lead to high BDQ MICs have only been found in laboratory-selected *Mtb* isolates (Huitric et al., 2010) while the first clinical isolates from patients treated with BDQ have showed a mixed pattern with MICs above and below the EUCAST break point (Zimenkov et al., 2017). Meanwhile several other genetic loci have been described, most notably mutations in the *Rv0678* gene that has been postulated to confer “low level” resistance to both BDQ and clofazimine (CFZ) and codes for a drug efflux pump regulator in *Mtb* (Andries et al., 2014). Mutations in the gene *pepQ* (*Rv2535*) (Almeida et al., 2016) has also been reported to encode determinants that lead to a low level MIC increase in BDQ and CFZ while mutations in *Rv1979c* (Zhang et al., 2015) only confers CFZ resistance. These findings have resulted in both excitement and confusion. These putative resistance determinants have been referred to as “resistance associated variants” or RAVs. Additional concerns have been raised on the potential for cross-resistance between BDQ and CFZ (Andries et al., 2014, Hartkoorn et al., 2014, Somoskovi et al., 2015, Xu et al., 2017), an age-old drug still used in leprosy which has recently moved to front line therapy for rifampicin-resistant (RR)/MDR TB, with WHO endorsement of the short-course regimen (WHO, 2016).

Our study sought to determine BDQ epidemiological cut-off values (ECVs) denoting resistance and thereby define interpretive criteria for phenotypic drug susceptibility testing (DST) results for the following methods: Middlebrook 7H10 agar dilution (M10A), Middlebrook 7H9 broth microdilution using the Thermo Fisher frozen microtiter plates (BMD) and the MGIT960 (MGIT). We also sought to assess the criteria against putative resistance associated variants (RAVs), microbiological outcomes and cross resistance with clofazimine (CFZ).

## 2 Materials and Methods

A retrospective cohort study was conducted using a broadly representative sample of *Mtb* isolates of varying resistance profiles from patients across South Africa. A BDQ naïve group was used to determine the wild-type distribution using all available RR-TB isolates from national drug resistance survey (NICD, 2016). The Clinical Laboratory Standards Institute (CLSI) recommends a sample size of > 300 isolates for ECV determination (CLSI, 2008). As the number was below what was required, additional isolates were included from routine drug resistance surveillance programs (GERMS-SA, 2015) in the country. The provincial distribution of isolates is shown in Fig. S1. A BDQ exposed group was used for comparison. These included patients on BDQ therapy having an elevated MIC on baseline testing on any method tested. The study was approved by the Human Research Ethics Committee of the University of the Witwatersrand, Johannesburg, South Africa under R14/49 and M160667.

The ECV study methods were designed in accordance with the CLSI M23-A3 guidance document: “Development of *in vitro* susceptibility criteria and quality control parameters” (CLSI, 2008). *Mtb* complex isolates, confirmed with the TBcID antigen test (Becton Dickinson, USA) and purity tested, we recovered on Middlebrook 7H10 agar and MGIT for MIC testing on M10A/BMD and MGIT respectively. BDQ MIC was performed by the M10A and BMD methods as previously described (Lounis et al., 2016, Kaniga et al., 2016). CFZ MICs were performed on BMD only following the same methodology as was done for the BDQ MIC on BMD. Custom-made microtiter plates were prepared by Thermo Fisher Scientific (Oakwood Village, Ohio, USA). The MIC for the M10A and BMD methods was defined as the lowest concentration of the drug-containing plate or well respectively, with no visual growth. The batch results were valid only if the H37Rv control fell within the published QC range (Lounis et al., 2016). The BACTEC™ MGIT™ 960 DST methods were followed as previously described (Torrea et al., 2015, Rusch-Gerdes et al., 2006) with slight modification to allow specific MICs to be tested for BDQ (Supplementary Information Box 1). The EpiCentre TBExist software (Becton Dickinson, USA) was used for interpretation of MIC for this method, and the incubation period extended from the recommended 13 to 28 days, adjusted for slow growing drug resistant isolates. The MGIT MIC was defined as the lowest concentration of drug-containing tube reported having a Growth Unit < 100.

WGS was performed using the MiSeq (Illumina, UK). Library preparation was performed using the Nextera-XT library preparation kit (Illumina, UK) and sequencing performed using the 2 × 300 bp MiSeq cartridge v.3 (Illumina, UK) with a target of 30 ×-50 × paired coverage (~ 80–100X coverage). CLC Genomics Workbench 8.5.1 was used to detect RAVs within the genes *atpE*, *pepQ, Rv1979c* and *Rv0678* using Reference mapping against the annotated reference genome H37Rv (NC00962.3) and the quality-based variant analysis tools.

The epidemiological cut-off value (ECV) was defined as the upper limit of the MIC value that separates the wild-type from the non-wild-type population (Schon et al., 2009, Turnidge and Paterson, 2007). An ECV of 95% (ECV_95_) was deemed susceptible (S) and an ECV between 95%–99.9% (ECV_99.9_) was deemed intermediate (I). For the range of dilutions used in the study, the frequency and cumulative frequency of MIC distribution were calculated for all three methods. The mode for each method was inspected against H37Rv QC range and if it differed by more than one dilution, the set was excluded (EUCAST, 2017) as this would imply variability in testing and skew the ECV. Histograms of the MIC distribution by each method were generated and the wild-type ECV estimated by iterative non-linear regression on expanding subsets (Turnidge et al., 2006) using the ECOFF finder tool (Turnidge and Paterson, 2007) and additional visual inspection. In addition, MIC ranges, MIC_90_ and MIC_95_ tables were generated. The MICs and associated ECVs were evaluated against the putative genes reported to encode BDQ resistance as well the MIC and RAV data of isolates from patients on BDQ based regimens. Lastly, the BDQ and CFZ MICs were cross tabulated to assess cross resistance and a Pearson's correlation coefficient determined.

## 3 Results

A total of 401 unique clinical isolates were tested in this study, 387 were from BDQ naïve patients and 14 from BDQ exposed patients. Of the 387, 9 were excluded due to loss of viability of the isolate, contamination or technical error. Of the remaining 378 BDQ naïve isolates, 310 (82%) isolates were RR/MDR and 68 (18%) were rifampicin susceptible. Among the RR/MDR, 97 (31%) isolates had a pre-XDR or XDR phenotype i.e. resistance to either a second-line injectable agent or a fluoroquinolone or both. Phenotypic BDQ MIC testing was performed for all 378 clinical isolates using the M10A, BMD and MGIT methods. Of the 14 BDQ exposed patients isolates, these were tested by the three phenotypic methods and WGS, with 1 isolate excluded due to loss of viability. Thus the analysable isolates were 378 BDQ naïve and 13 BDQ exposed with a final total of 391 isolates.

**Fig. 1:**
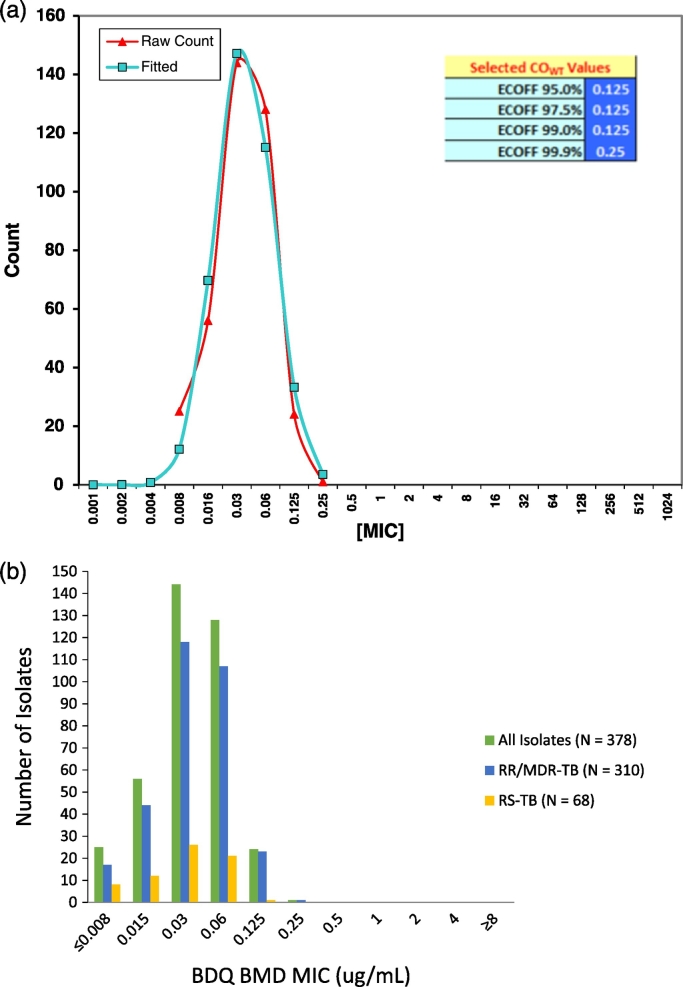
a: Wild-type ECV estimation using iterative non-linear regression on expanding subsets for BDQ on BMD (N = 378). b: Histogram of BDQ BMD MIC distribution (μg/ml), N = 378.

**Fig. 2:**
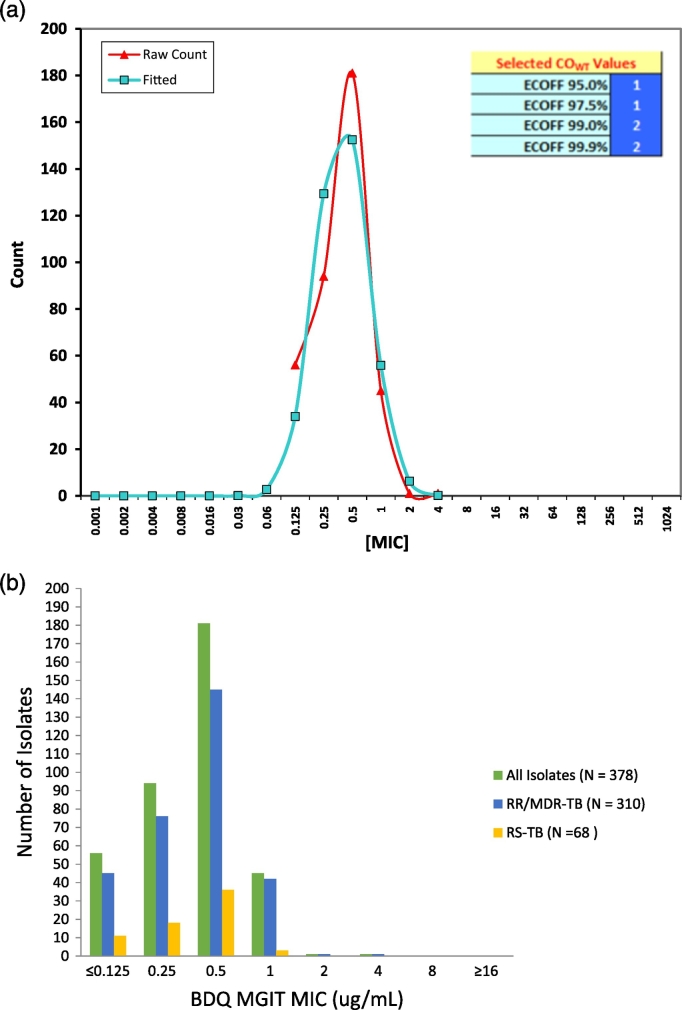
a: Wild-type ECV estimation using iterative non-linear regression on expanding subsets for BDQ on MGIT (N = 378). b: Histogram of BDQ MGIT MIC distribution (μg/ml), N = 378.

The MIC distributions for all three phenotypic methods are shown as histograms in Figs. 1b, 2b and S2b. The mode for both BMD and MGIT were in line with the H37Rv distribution, however the mode for M10A was 0.25 μg/ml which was more than one dilution above that for H37Rv (0.06 μg/ml) on the same method (Tables S1a, b and S2) and the data for this method was excluded from the primary analysis. The ECV estimation using iterative non-linear regression on expanding subsets for BDQ on BMD and MGIT are shown in Figs. 1a, 2a and S2a with both actual and fitted values shown. Further, a unimodal distribution was observed for these methods and the calculated ECVs at different thresholds embedded in these figures. The ECV_95_ was 0.125 μg/ml and 1 μg/ml respectively for BMD and MGIT and MICs less than or equal to ECV_95_ were categorised as susceptible. The ECV_99.9_ was 0.25 μg/ml and 2 μg/ml for BMD and MGIT, respectively and isolates at these MICs deemed intermediate. An MIC above the ECV_99.9_ was categorised as resistant.

The MIC_90_ and MIC_95_ are shown in Table 1 and disaggregated by resistance type. The MIC_95_ values are lower or equal to the ECV_95_ for all resistance subtypes, further supporting the selection of the ECVs to clearly delineate the wild-type and non-wild-type populations. The CFZ ECV_95_ on BMD was determined to be 0.25 μg/ml and the ECV_99.9_ was 0.5 μg/ml (Fig. S3).

**Table 1:** BDQ susceptibility profile by resistance type and testing method.

DST	Resistance	MIC (μg/ml)			
Method	Subtypes	N	MIC range	MIC_90_	MIC_95_
BMD	All isolates	378	≤ 0.008–0.25	0.06	0.125
BMD	RR/MDR-TB	310	≤ 0.008–0.25	0.06	0.125
BMD	RS-TB	68	≤ 0.008–0.125	0.06	0.06
MGIT	All isolates	378	≤ 0.125–4	1	1
MGIT	RR/MDR-TB	310	≤ 0.125–4	1	1
MGIT	RS-TB	68	≤ 0.125–1	0.5	0.5

RS: rifampicin susceptible, RR: rifampicin resistant, MDR: multidrug resistant.

Whole genome sequencing was successful for 377/378 (> 99%) of the isolates with a depth of coverage ranging between 22X and 64X paired coverage (~ 44–128X). The impact of RAVs on ECVs was assessed by determining the frequency distribution of RAVs across MICs as a form of additional validation (Table 2, Table 3 and S3a,b). Scattered RAVs across the BDQ MIC range would indicate questionable relevance of the variants while RAVs centred at one MIC would warrant an adjustment of the ECV for the DST methods evaluated in this study. No *atpE* mutations were detected while 3 (0.8%), 4 (1.1%) and 98 (25.9%) mutations were observed in the *Rv0678, pepQ* and *Rv1979* genes respectively. Of the three *Rv0678* RAVs identified, two were in the susceptible category and one RR/MDR case was intermediate for both BMD and MGIT. All of the isolates with mutations in *pepQ* (*Rv2535c*) were observed in the susceptible category for both methods. Among the *Rv1979* RAVs, ≤ 1% was above the intermediate category for BMD and MGIT.

**Table 2:** Putative RAVs and MIC distribution tested on BMD among BDQ naïve isolates.

Mutations	BDQ BMD MIC (mg/ml)											Total
	Susceptible					Intermediate	Resistant					
	≤ 0.008	0.015	0.03	0.06	0.125	0.25	0.5	1	2	4	≥ 8	
*atpE*												0
*Rv0678*				1	1	**1**						3
*pepQ (Rv2535)*			2	1	1							4
*Rv1979*	2	10	31	41	14							98

**Table 3:** Putative RAVs and MIC distribution tested on MGIT among BDQ naïve isolates.

Mutations	BDQ MGIT MIC (mg/ml)								Total
	Susceptible				Intermediate	Resistant			
	≤ 0.125	0.25	0.5	1	2	4	8	≥ 16	
*atpE*									**0**
*Rv0678*			1	1	1				**3**
*pepQ (Rv2535)*			2	2					**4**
*Rv1979*	6	23	59	9		1			**98**

The MIC results stratified by microbiological outcome for the 13 patients with prior BDQ exposure unrelated to the BDQ naïve patients are shown in Table 4. Isolates for these cases prior to BDQ treatment were unavailable for DST. Among patients that culture converted by month 6, all had MICs in the susceptible category by either BMD or MGIT and the number of susceptible companion drug classes ranged between 3 and 6. Among the other 8 patients who remained culture positive at month 6 or later, all had MICs at or above the intermediate category by BMD and 6 of these cases had an *Rv0678* mutation. Two of these cases shared the same mutation (141_142insC). The number of drug classes showing susceptibility ranged between 1 and 2. Interestingly, of the 13 patient isolates, two had a MGIT MIC of 1 μg/ml deemed susceptible. The one had culture converted at 6 months while the other did not, with the notable difference being the number of companion susceptible drugs, 3 and 1 respectively and the absence or presence of an *Rv0678* mutation.

**Table 4:** Characteristics of BDQ exposed patient isolates.

Month^a^	Sub-type	Genetic targets for BDQ				Genome coverage	BDQ MGIT	BDQ BMD	INH	RMP	LZD	CFZ	LVX	OFX	MXF	CAP	KAN	AMI	EMB	No. susceptible drug classes
		*atpE*	*Rv0678*	*pepQ*	*Rv1979*															
Cultured negative at month 6																				
2	XDR	wt	wt	wt	wt	60.6	0.25	0.06	***16***	**> 4**	1	0.25	**> 4**	***8***	***4***	***4***	***16***	2	***16***	4
2	Pre-XDR	wt	wt	wt	wt	61.0	0.25	0.03	***> 16***	**> 4**	2	0.125	***4***	***8***	2	2	4	1	4	6
2	XDR	wt	wt	wt	G1226A	49.4	0.25	0.125	***> 16***	**> 4**	1	0.125	***4***	***8***	2	***16***	***> 16***	***> 16***	***8***	4
0	XDR	wt	wt	wt	G1226A	63.2	0.5	0.06	***16***	**> 4**	0.5	0.125	***4***	***8***	***4***	***16***	***> 16***	***> 16***	***8***	3
2	XDR	wt	wt	wt	G1226A	55.3	1	0.06	***> 16***	**> 4**	1	0.25	***> 4***	***> 8***	***4***	***16***	***> 16***	***> 16***	***8***	3

Culture positive at month 6 or later																				
6 +	XDR	wt	wt	wt	G1226A	55.8	**2**	**0.25**	***16***	***> 4***	***8***	***0.5***	***4***	***8***	1	***16***	***> 16***	***> 16***	***16***	1
6 +	XDR	wt	136_137insG	wt	G1226A	54.5	**2**	**0.25**	***16***	***> 4***	0.5	***0.5***	***4***	***> 8***	***4***	***8***	***> 16***	***> 16***	***8***	1
6 +	XDR	wt	wt	wt	G1226A	55.4	**2**	***0.5***	***> 16***	***> 4***	1	***2***	***> 4***	***> 8***	***> 4***	***16***	***> 16***	***> 16***	***16***	1
6 +	XDR	wt	138_139insG	wt	G1226A	56.1	**2**	***0.5***	***> 16***	***> 4***	2	***2***	***> 4***	***> 8***	***> 4***	***> 16***	***> 16***	***> 16***	***> 16***	1
6 +	XDR	wt	141_142insC	wt	G1226A	55.3	***4***	**0.25**	***16***	***> 4***	1	***1***	***> 4***	***> 8***	***4***	***16***	***> 16***	***> 16***	***8***	1
6 +	XDR	wt	T200G	wt	G1226A	55.1	***4***	***0.5***	***4***	***> 4***	2	***2***	***2***	***8***	***4***	***4***	2	1	***> 16***	2
6 +	XDR	wt	345delG	wt	G1226A	55.6	***4***	***0.5***	***> 16***	***> 4***	1	***1***	***> 4***	***> 8***	***4***	***16***	***> 16***	***> 16***	***16***	1
6 +	XDR	wt	141_142insC	wt	G1226A	62.9	1	***0.5***	***16***	***> 4***	***8***	***0.5***	***> 4***	***> 8***	***> 4***	***> 16***	***> 16***	***> 16***	***8***	1

INH: isoniazid, RMP: rifampicin, LZD: linezolid, CFZ: clofazimine, OFX: ofloxacin, MXF: moxifloxacin, CAP: capreomycin, KAN: kanamycin, AMI: amikacin, EMB: ethambutol.

Bold and italic = resistant, bold = intermediate.

a Month: on treatment when culture isolate tested. For 1 case the patient had a baseline isolate (month 0) but had a previous BDQ treatment episode.

**Fig. 3:**
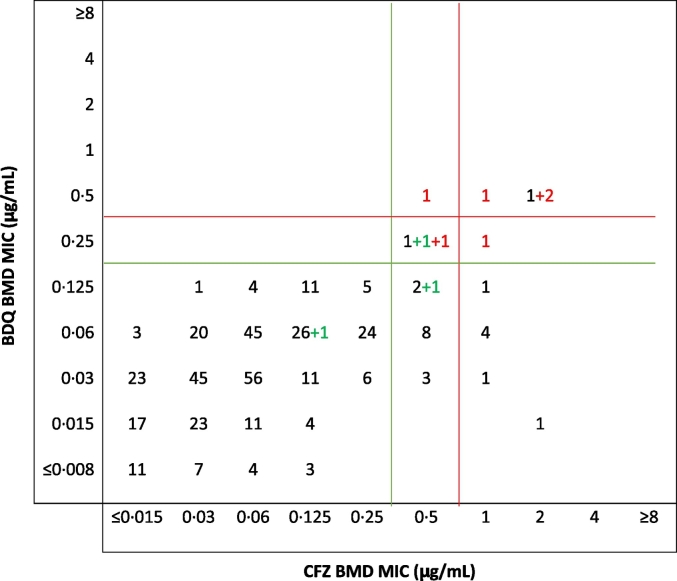
Cross tabulation of BDQ and CFZ MICs (N = 391). Numbers in red are *Rv0678* RAVs from BDQ exposed patient isolates while the numbers in green are *Rv0678* RAVs from BDQ naïve patients. Numbers in black are wild type for *Rv0678* and BDQ naïve. Green line: ECV 95%, Red line: ECV 99.9%.

Using the ECV_99.9_ determined for BDQ and CFZ on BMD to define resistance, a tentative inference was drawn about cross-resistance between the two antimicrobial agents in study isolates from regression analyses comparing their MICs on the same scale (CLSI, 2008). If cross-resistance existed, a strong correlation between the MICs of the two agents was expected and a majority of the MICs would be clustered around a 45-degree diagonal. Among the 391 BDQ naïve and exposed with complete results (Fig. 3), there was a strong correlation (|* r* | = 0.5; *p* < 0.001) between BDQ and CFZ MICs. The presence of an *Rv0678* RAV showed an even stronger correlation (|* r* | = 0.8; *p* = 0.007).

Five (42%) of 12 CFZ-resistant isolates were BDQ-resistant/intermediate (Table S4). Four (22%) of 18 CFZ-intermediate isolates were BDQ-resistant/intermediate. None (0%) of 361 CFZ-susceptible isolates were BDQ-resistant/intermediate. In contrast, all 9 (100%) of the BDQ-resistant/intermediate isolates were CFZ-resistant/intermediate, whereas only 21 (5.5%) of 382 BDQ-susceptible isolates were CFZ-resistant/intermediate. Of the 13 CFZ-resistant isolates, 4 (31%) had an *Rv0678* mutation. All 4 of these mutants were resistant or only intermediately susceptible to BDQ and were isolated from patients with prior BDQ exposure. Of the 18 CFZ-intermediate isolates, 4 (22%) had an *Rv0678* mutation. Two of these 4 mutants were isolated from BDQ-exposed patients and were either resistant or intermediately susceptible to BDQ. The other 2 CFZ-intermediate *Rv0678* mutants were isolated from BDQ-naïve patients: one was intermediately susceptible to BDQ and the BDQ MIC for the other was 0.125 μg/ml. Thus, among all 9 mutants identified, 8 were resistant or intermediately susceptible to CFZ, and 7 of these were resistant or intermediately susceptible to BDQ while the other one had an MIC exceeding BDQ MIC_90_ for the collection. All CFZ-susceptible isolates were susceptible to BDQ. Among the three BDQ naïve patient isolates with *Rv0678* RAVs, one was intermediate to both drugs and harboured a A152C polymorphism in the *Rv0678* gene. The mutations for the other two BDQ naïve patient isolates were C158T and 141_142insC, and susceptible to both drugs. The latter mutation occurred in a patient with primary MDR-TB; however, the same mutation also occurred in 2 other cases with BDQ exposure, which showed resistance to both CFZ and BDQ.

## 4 Discussion

The introduction of BDQ, a new class of anti-mycobacterial agent introduced after several decades, has been welcomed with great enthusiasm. This, along with another new drug – delamanid – has reversed the dire prognosis of many patients with XDR-TB who have had limited therapeutic options. However, this temporary reprieve may be short lived if emergence of resistance to these new agents is not identified early and the void in susceptibility testing closed.

We conducted the largest study to date defining interpretive criteria for BDQ susceptibility as they apply to the most common phenotypic methods in use. Defining interpretive criteria for TB is often challenging as patients are on multiple drug regimens and assessing clinical outcome related to a single drug is often not possible. For clinical break-point setting, ECVs are widely used and provides an important basis for defining susceptibility. Information on pharmacokinetic and pharmacodynamics is also important but data is usually sparse and influenced by host co-morbidities, genetics and several other factors. It is even more challenging when the genetic basis for resistance is not fully understood.

The WHO critical concentrations for *Mtb* has historically used 95% of the wild type population as criterion to define susceptibility (Angeby et al., 2012). This by definition allows for errors in classification, and EUCAST and FDA instead use the upper limit of the wild type distribution. In the current study, we have defined isolates as susceptible and intermediate using criteria which correlate with the WHO and EUCAST/FDA criteria and were 0.125 μg/ml and 0.25 μg/ml for BMD and 1 μg/ml and 2 μg/ml for MGIT respectively. This provides a robust approach and allows greater certainty in interpretation and detection of isolates with RAVs.

The EUCAST set a criterion of ≤ 0.25 μg/ml to define susceptibility using the BMD method. The EUCAST decision was based on data that used BDQ MICs from baseline *Mtb* isolates from subjects in clinical trials C208 Stage2 and C209 and their sputum culture conversion rates at Week 24 (FDA, 2012). In that analysis, 3 of 4 patients tested on the resazurin microtiter assay (REMA) – a non-commercial BMD – had culture converted at week 24 with an MIC of 0.25 μg/ml while the single patient with an MIC of 0.5 μg/ml failed to culture convert. In the current study, of the 13 patients on a BDQ based regimen with a 6-month culture conversion outcome (Table 4), three had an MIC of 0.25 μg/ml on BMD and all three failed to convert. However, it is not possible to conclude that these isolates were truly resistant as the number of available drug classes showing susceptibility was only one in the majority of non-converters and the pre-treatment BDQ MICs are unknown. Thus, defining such cases as intermediate is appropriate and would concur with the clinical trial data.

Furthermore, although we cannot at this stage define clinical breakpoints, pharmacokinetic data from a study in SA showed a steady state concentration for BDQ at week 8 on combination therapy of 902 ± 535 ng/L ml (~ 0.902 ± 0.535 μg/ml) (Diacon et al., 2009). Thus it is possible that isolates in the intermediate category (0.25 μg/ml) may achieve killing in vivo. However, these values are total drug concentrations whereas the drug is highly protein-bound and complicates interpreting the presumed in-vivo activity. Furthermore, the average concentration has been reported to drop to 0.58μg/ml between weeks 8 and 24 (van Heeswijk et al., 2014), thus clinical studies combining PK/PD and MIC determinations are required to fully define clinical break points.

Applying the EUCAST provisional criteria to testing on MGIT would however result in 60% (228/378) of isolates being false resistant, thus method specific criteria are required. Our findings are similar to the study by Torrea et al. (Torrea et al., 2015) who also determined 1 μg/ml as the ECV for MGIT though a limited number of MDR-TB isolates, while a separate study by Keller and colleagues (Keller et al., 2015) using crushed tablets determined the value to be 1.6 μg/ml. Unlike the microtitre assays that determine MICs, MGIT is conventionally designed to test a single concentration and testing BDQ at 1 μg/ml would be appropriate given the low levels of resistance currently. It is however not possible to conclude that an MIC of 2 μg/ml could be directly linked to failure. Thus, growth observed at 1 μg/ml should be reconfirmed with testing at 2 μg/ml. In addition, and where available, sequencing of the *Rv0678* gene should be performed to determine the potential risk for resistance development. Interestingly, two patients had a BDQ MIC of 1 μg/ml on MGIT with one converting at 6 months and the other not; the key difference is that the former had three drug classes being susceptible including BDQ while the latter was essentially on BDQ monotherapy. The higher ECVs determined for MGIT versus the BMD method is unknown, but may be explained by adsorption of the drug to the type of plastic-ware applied in the tests. BMD used polystyrene microtitre plates while polycarbonate tubes are used in MGIT. The degree of adsorption on polycarbonate is unknown.

The genetic basis of resistance to BDQ is still the subject of much uncertainty. Early studies highlighted the role of RAVs in the *atpE* gene, leading to resistance. However, this was only demonstrated by in-vitro selection studies (Huitric et al., 2010) until very recently (Zimenkov et al., 2017), and none were found in the current study. Mutations in the gene *pepQ,* reported to confer low-level BDQ and CFZ resistance (Almeida et al., 2016, Zhang et al., 2015), together with RAVs in the gene *Rv1979,* recently reported to confer CFZ resistance, have emerged as potentially relevant (Zhang et al., 2015). All isolates with the RAVs in *pepQ* were susceptible only 1% of the *Rv1979* RAVs were resistant. Thus the concept of “low-level resistance” may be a misnomer as criteria defining resistance in the past were not clearly defined and what was actually described is MICs near the upper-bound of the wild-type distribution.

Mutations in *Rv0678* have generated the most interest and shown to result in variable expression of the MmpS5-MmpL5 efflux pump with similar variability in increasing MICs to BDQ (Andries et al., 2014). A total of nine *Rv0678* RAVS were found in this study (Fig. 3) and among 3 BDQ naïve patients none were resistant, 1 was intermediate and 1 was at the MIC_90_ by BMD while in those with prior BDQ exposure, all six were resistant or intermediate. Three of the nine had the same mutation (141_142insC), the BDQ naïve isolate was susceptible (0.06 μg/ml) and the two BDQ exposed showed resistance by at least one method suggesting that exposure may trigger activation of the pump.

A study by Zimenkov and colleagues from Russia (Zimenkov et al., 2017) reported a case with a baseline *Rv0678* RAV who subsequently acquired an *atpE* RAV on BDQ therapy, and this dual mutant had the highest MIC of all those reported in that study. Thus, although the MICs for *Rv0678* RAVs may only lead to elevations close to the ECV, these may serve as step-mutations leading to further resistance. A study analysing data from MDR-TB clinical trials noted a high prevalence of these RAVs (6.3%) in patients without past exposure to either BDQ or CFZ (Villellas et al., 2017). In our study with a more generalizable population we found a lower prevalence of 1.4% (3/213) among BDQ naïve RR/MDR cases, and none among rifampicin susceptible cases (0/68).

Mutations in *Rv0678* per se may not result in high MICs, but drug exposure would result in elevated MICs above the ECV. Further studies to assess in-vitro drug exposure levels and their potential impact on emergence of resistance would be important. Spread of resistance is likely to occur once these mutations are established and preventing its occurrence will be essential. Among those with prior BDQ exposure and who had culture converted by month 6, none had an *Rv0678* mutation. These patients had at least three companion drugs suggesting that selective amplification of efflux pump mutant sub-populations or mutagenesis itself may be preventable when effective combination therapies were applied. Thus, comprehensive baseline testing is essential and avoidance of the use of a new drug when at least three effective supporting drugs cannot be guaranteed. Additionally, empiric use of combination regimens containing the new drugs for pre-XDR-TB and XDR-TB with little background resistance is recommended.

An emerging concern is the potential for cross-resistance between CFZ and BDQ (Hartkoorn et al., 2014). A strong correlation was observed between BDQ and CFZ MICs and was even stronger when a *Rv0678* RAV was present. This suggests that they probably share a common biochemical pathway, cross resistance occurs and is particularly notable for isolates that have a *Rv0678* RAV. Among CFZ intermediate/resistant isolates 30% (9/30) were also intermediate/resistant to BDQ while among BDQ resistant isolates all 9 (100%) were resistant to CFZ with the majority (8/9) harbouring an *Rv0678* RAV. Thus BDQ resistance likely confers complete cross resistance to CFZ while among those with CFZ resistance a third would be cross resistant. Thus, other mechanisms are dominant causes of CFZ resistance and do not lead to BDQ resistance. Clofazimine has primarily been used to treat XDR-TB in South Africa and in the current study, all of the pre-XDR and XDR cases that were BDQ naïve showed BDQ susceptibility and none harboured an *Rv0678* mutation. Use of this repurposed drug is set to change, following the recent WHO recommendations to use the 9 month regimen (WHO, 2016), with CFZ as one of the core drugs, for all RR/MDR-TB cases. Ongoing monitoring will be important as the occurrence of these RAVs may increase over time with the policy shift which has potential implications for BDQ. Neither prior CFZ exposure nor resistance predicts BDQ resistance, thus these patients could receive BDQ and susceptibility testing to BDQ should be used for resistance determination.

The current study has provided important new data on BDQ. Although this was not a multi-country study, the findings have been in keeping with published literature from other countries. In addition, the current work will now form the basis of a WHO multi-country validation study for BDQ susceptibility testing and a multi-country surveillance program. The exclusion of M10A from further analysis was unfortunate and reasons for the variance were investigated but no technical issues could explain the results. Variability was also noted in the original QC study (Kaniga et al., 2016) and the study by Zimenkov (Zimenkov et al., 2017) and further investigations on the reproducibility of this method are warranted. The isolates included in the BDQ exposed group was small and selected based on initial high MICs. This is a potentially biased group towards BDQ resistance but was purposefully chosen to allow comparison with the BDQ naïve group.

Our findings provide robust criteria that should facilitate routine phenotypic drug susceptibility testing for BDQ and also stimulate further research. The number of patients requiring a BDQ based regimen following failure on the 9-month CFZ based regimen are likely to increase and BDQ testing, preferably using the MGIT methodology, should be performed in all cases. Rapid molecular testing would be preferred, but at this stage, does not seem feasible as RAVs do not appear to be concentrated in genomic hotspots. In addition, genes related to resistance have not been fully elucidated and require further research, as some proposed genes have not been found to be linked to resistance in this study. Baseline BDQ resistance does not appear to be common and emergence of resistance can be prevented by ensuring that supporting drugs in the regimen are effective.

## Funding Sources

Janssen Pharmaceuticals provided funding for consumables used in the study. All study procedures, data collection and analyses were independently conducted by the WHO Supranational TB Reference Laboratory at the Centre for Tuberculosis (CTB), the National Institute for Communicable Diseases (NICD) in Johannesburg, South Africa. All authors assure the accuracy and completeness of the data reported. The corresponding author had full access to all the data in the study and had final responsibility for the decision to submit for publication.

## Conflict of Interests

KK is an employee of Janssen Pharmaceuticals. All other authors declare no competing interests.

## Authors' Contributions

NAI, SV, AWD, HK, KK and NN were involved in the conception and design of the study. NAI, SV, LJ, NG, LB, FI, AWD, HK, KK and NN were involved in study implementation. NAI, SV and LB did the data analysis. NAI, SV, AWD, HK, KK and NN interpreted the data and provided important intellectual input. NAI, SV, FI, HK, KK and NN wrote the first draft.

## Appendix A Supplementary data

Supplementary material Image 1
