# Supplementary material for: Defining Bedaquiline Susceptibility, Resistance, Cross-Resistance and Associated Genetic Determinants: A Retrospective Cohort Study
Source: eBioMedicine. 2018 Jan 9;28:136–42. doi: 10.1016/j.ebiom.2018.01.005 (PMC5835552; doi:10.1016/j.ebiom.2018.01.005)
Supplement: Supplementary file 1 — Supplementary material [file mmc1.docx]

**Supplementary material**

**TITLE: Defining Bedaquiline susceptibility, resistance, cross-resistance and associated genetic determinants: a retrospective cohort study**

**AUTHORS:**

*Nazir A Ismail^1,2^, *Shaheed V Omar^1^, Lavania Joseph^1^, Netricia Govender^1^, Linsay Blows^1^, Farzana Ismail^1,2^, Hendrik Koornhof^1^, Andries W Dreyer^1^, Koné Kaniga^3^, Norbert Ndjeka^4^

^1^National Institute for Communicable Diseases, Centre for Tuberculosis, Johannesburg, South Africa

^2^Department of Medical Microbiology, University of Pretoria, Pretoria, South Africa

^3^Janssen Research & Development, Titusville, NJ, United States of America

^4^National Department of Health, Tuberculosis Control and Management Cluster, Pretoria, South Africa

Corresponding author:

Dr Nazir Ahmed Ismail

Centre for Tuberculosis, National Institute for Communicable Diseases,

A division of the National Health Laboratory Service

1 Modderfontein Road, Sandringham, Johannesburg 2131, South Africa

Office: +27-11-8855-321

Cell: +27-82-6000-857

Email: [naziri@nicd.ac.za](mailto:naziri@nicd.ac.za)

Table S1a: BDQ MIC distribution pattern by test method type

| **Test method** | **Type of isolate** | **Number of isolates** | **BDQ MIC (μg/L)** | | | | | | | | | | | |
| --- | --- | --- | --- | --- | --- | --- | --- | --- | --- | --- | --- | --- | --- | --- |
|  |  |  | **≤0·008** | **0·015** | **0·03** | **0·06** | **0·125** | **0·25** | **0·5** | **1** | **2** | **4** | **8** |  |
| M10A | H37Rv ATCC 27294 | 34 |  |  | 6 | **28** |  |  |  |  |  |  |  |  |
| M10A | Clinical | 378 |  | 2 | 9 | 70 | 95 | **142** | 60 |  |  |  |  |  |
| BMD | H37Rv ATCC 27294 | 31 |  |  | 14 | **17** |  |  |  |  |  |  |  |  |
| BMD | Clinical | 378 | 25 | 56 | **144** | 128 | 24 | 1 |  |  |  |  |  |  |

Numbers in bold indicate mode of MIC distribution, N/A: not applicable

Table S1b: BDQ MIC distribution pattern by test method type

| **Test method** | **Type of isolate** | **Number of isolates** | **BDQ MIC (μg/L)** | | | | | | | | | | | |
| --- | --- | --- | --- | --- | --- | --- | --- | --- | --- | --- | --- | --- | --- | --- |
|  |  |  | **≤0·008** | **0·015** | **0·03** | **0·06** | **≤0·125** | **0·25** | **0·5** | **1** | **2** | **4** | **8** |  |
| MGIT | H37Rv ATCC 27294 | 24 |  |  |  |  | 4 | **11** | 7 | 2 |  |  |  |  |
| MGIT | Clinical | 378 |  |  |  |  | 56 | 94 | **181** | 45 | 1 | 1 |  |  |

Numbers in bold indicate mode of MIC distribution, N/A: not applicable

Table S2: BDQ susceptibility profile by resistance type by 7H10 agar

| DST | Resistance |  | MIC (µg/mL) | | |
| --- | --- | --- | --- | --- | --- |
| Method | Subtypes | N | MIC Range | MIC_90_ | MIC_95_ |
| BDQ 7H10 Agar | All Isolates | 378 | 0.015-0.5 | 0.5 | 0.5 |
| BDQ 7H10 Agar | MDR-TB | 310 | 0.015-0.5 | 0.5 | 0.5 |
| BDQ 7H10 Agar | DS-TB | 68 | 0.03-0.5 | 0.5 | 0.5 |

Table S3a: Wild type and putative mutants with MIC distribution tested on BMD among BDQ naïve isolates (N=378)

|  |  | **BDQ BMD MIC (ug/mL)** | | | | | | | | | | |  |
| --- | --- | --- | --- | --- | --- | --- | --- | --- | --- | --- | --- | --- | --- |
|  |  | **Susceptible** | | | | | **Intermediate** | **Resistant** | | | | | **Total** |
|  |  | ≤0.008 | 0.015 | 0.03 | 0.06 | 0.12 | 0.25 | 0.5 | 1 | 2 | 4 | ≥8 |  |
| *atpE* | *mutant* |  |  |  |  |  |  |  |  |  |  |  | 0 |
|  | *wild type* | 25 | 55 | 144 | 128 | 24 | 1 |  |  |  |  |  | 377 |
| *Rv0678* | *mutant* |  |  |  | 1 | 1 | 1 |  |  |  |  |  | 3 |
|  | *wild type* | 25 | 55 | 144 | 127 | 23 |  |  |  |  |  |  | 374 |
| *pepQ (Rv2535)* | *mutant* |  |  | 2 | 1 | 1 |  |  |  |  |  |  | 4 |
|  | *wild type* | 25 | 55 | 142 | 127 | 23 | 1 |  |  |  |  |  | 373 |
| *Rv1979* | *mutant* | 2 | 10 | 31 | 41 | 14 |  |  |  |  |  |  | 98 |
|  | *wild type* | 23 | 45 | 113 | 87 | 10 | 1 |  |  |  |  |  | 279 |

Table S3b: Wild type and putative mutants with MIC distribution tested on MGIT among BDQ naïve isolates (N=378)

|  |  | **BDQ MGIT 960 MIC (ug/mL)** | | | | | | | |  |
| --- | --- | --- | --- | --- | --- | --- | --- | --- | --- | --- |
|  |  | **Susceptible** | | | | **Intermediate** | **Resistant** | | | **Total** |
|  |  | ≤0.12 | 0.25 | 0.5 | 1 | 2 | 4 | 8 | ≥16 |  |
| *atpE* | *mutant* |  |  |  |  |  |  |  |  | 0 |
|  | *wild type* | 56 | 93 | 181 | 45 | 1 | 1 |  |  | 377 |
| *Rv0678* | *mutant* |  |  | 1 | 1 | 1 |  |  |  | 3 |
|  | *wild type* | 56 | 93 | 180 | 44 |  | 1 |  |  | 374 |
| *pepQ (Rv2535)* | *mutant* |  |  | 2 | 2 |  |  |  |  | 4 |
|  | *wild type* | 56 | 93 | 179 | 43 | 1 | 1 |  |  | 373 |
| *Rv1979* | *mutant* | 6 | 23 | 59 | 9 |  | 1 |  |  | 98 |
|  | *wild type* | 50 | 70 | 122 | 36 | 1 |  |  |  | 279 |

Table S4: Cross tabulation of BDQ and CFZ drug susceptibility categories (N=391)

|  |  | Clofazimine | | | |
| --- | --- | --- | --- | --- | --- |
|  |  | S | I | R | Total |
| Bedaquiline | S | 360+1 | 13+1 | 7 | 382 |
|  | I | 0 | 1+1+1 | 1 | 4 |
|  | R | 0 | 1 | 3+1 | 5 |
|  | Total | 361 | 18 | 12 | 391 |

Numbers in red are *Rv0678* RAVs from BDQ exposed patient isolates while the numbers in green are *Rv0678* RAVs from BDQ naïve patients. Numbers in black are wild type for *Rv0678* and BDQ naïve.

Figure S1: Provincial distribution of BDQ naïve isolates (N=378)

Figure S2a: Wild-type ECV estimation using iterative non-linear regression on expanding subsets for M10A (N=378)

Figure S2b: Histogram of BDQ M10A MIC distribution (μg/ml), N=378

Figure S3: Wild-type ECV estimation using iterative non-linear regression on expanding subsets for BMD for CFZ (N=378)

Figure S4: Wild-type ECV estimation using iterative non-linear regression on expanding subsets for BMD for LZD (N=378)

Supplementary Information Box 1

Two-fold serial dilutions of 100X working solutions of BDQ were prepared from a 1 mg/ml stock solution using dimethylsulfoxide (DMSO) and aliquots were kept no longer than 3 months at -70°C. The 100X working solutions in DMSO were further diluted in the MGIT tubes to obtain final concentrations of 8, 4, 2, 1, 0·5, 0·25, 0·125 μg/mL. The bacterial suspensions were prepared from MGIT subcultures and used for DST inoculation within the specified time.
